# Intention to Use Wiki-Based Knowledge Tools: Survey of Quebec Emergency Health Professionals

**DOI:** 10.2196/24649

**Published:** 2021-06-18

**Authors:** Patrick Archambault, Stéphane Turcotte, Pascal Y Smith, Kassim Said Abasse, Catherine Paquet, André Côté, Dario Gomez, Hager Khechine, Marie-Pierre Gagnon, Melissa Tremblay, Nicolas Elazhary, France Légaré, Wiki-Based Knowledge Tool Investigators

**Affiliations:** 1 Département de médecine d'urgence Centre intégré de santé et de services sociaux de Chaudière-Appalaches Lévis, QC Canada; 2 Department of Family Medicine and Emergency Medicine Faculty of Medicine Université Laval Québec, QC Canada; 3 VITAM - Centre de recherche en santé durable Université Laval Québec, QC Canada; 4 Centre intégré de santé et de services sociaux de Chaudière-Appalaches Lévis, QC Canada; 5 Département de management Faculté des sciences de l’administration Université Laval Québec, QC Canada; 6 Département de marketing Faculté des sciences de l’administration Université Laval Québec, QC Canada; 7 Département de systèmes d’information organisationnels Faculté des sciences de l’administration Université Laval Québec, QC Canada; 8 Faculté des sciences infirmières Université Laval Québec, QC Canada; 9 Department of Family Medicine and Emergency Medicine Faculty of Medicine Université de Sherbrooke Sherbrooke, QC Canada; 10 see Acknowledgments

**Keywords:** knowledge management, knowledge translation, implementation science, collaborative writing applications, wikis, trauma care

## Abstract

**Background:**

Clinical decision support systems are information technologies that assist clinicians in making better decisions. Their adoption has been limited because their content is difficult to adapt to local contexts and slow to adapt to emerging evidence. Collaborative writing applications such as wikis have the potential to increase access to existing and emerging evidence-based knowledge at the point of care, standardize emergency clinical decision making, and quickly adapt this knowledge to local contexts. However, little is known about the factors influencing health professionals’ use of wiki-based knowledge tools.

**Objective:**

This study aims to measure emergency physicians’ (EPs) and other acute care health professionals’ (ACHPs) intentions to use wiki-based knowledge tools in trauma care and identify determinants of this intention that can be used in future theory-based interventions for promoting the use of wiki-based knowledge tools in trauma care.

**Methods:**

In total, 266 EPs and 907 ACHPs (nurses, respiratory therapists, and pharmacists) from 12 Quebec trauma centers were asked to answer a survey based on the theory of planned behavior (TPB). The TPB constructs were measured using a 7-point Likert scale. Descriptive statistics and Pearson correlations between the TPB constructs and intention were calculated. Multiple linear regression analysis was conducted to identify the salient beliefs.

**Results:**

Among the eligible participants, 57.1% (152/266) of EPs and 31.9% (290/907) of ACHPs completed the questionnaire. For EPs, we found that attitude, perceived behavioral control (PBC), and subjective norm (SN) were significant determinants of the intention to use wiki-based knowledge tools and explained 62% of its variance. None of the sociodemographic variables were related to EPs’ intentions to use wiki-based knowledge tools. The regression model identified two normative beliefs ("approval by physicians" and "approval by patients") and two behavioral beliefs ("refreshes my memory" and "reduces errors"). For ACHPs, attitude, PBC, SN, and two sociodemographic variables (profession and the previous personal use of a wiki) were significantly related to the intention to use wiki-based knowledge tools and explained 60% of the variance in behavioral intention. The final regression model for ACHPs included two normative beliefs ("approval by the hospital trauma team" and "people less comfortable with information technology"), one control belief ("time constraints"), and one behavioral belief ("access to evidence").

**Conclusions:**

The intentions of EPs and ACHPs to use wiki-based knowledge tools to promote best practices in trauma care can be predicted in part by attitude, SN, and PBC. We also identified salient beliefs that future theory-based interventions should promote for the use of wiki-based knowledge tools in trauma care. These interventions will address the barriers to using wiki-based knowledge tools, find ways to ensure the quality of their content, foster contributions, and support the exploration of wiki-based knowledge tools as potential effective knowledge translation tools in trauma care.

## Introduction

### Background

Emergency physicians (EPs) and other acute care health professionals (ACHPs), such as nurses, respiratory therapists, and pharmacists, working in fast-paced emergency departments (EDs) rely on heuristic clinical reasoning that can falter and lead to unconscious acts of omission and contribute to medical errors [1-4]. Overuse of diagnostic modalities has also become a major challenge, which exposes patients to unwarranted tests and procedures [5]. Clinical decision support systems (CDSSs) are health information technologies that have been proposed as solutions to assist clinicians in making better decisions [6]. These technologies are of great importance for knowledge management, organizational learning, and knowledge-building purposes in ways that allow decision making to be more productive, agile, innovative, and reputable [7]. Systematic reviews have found that CDSS can help professionals in implementing best practices [8,9] and be effective in promoting changes in a variety of clinical areas and environments [10-14]. CDSS may also reduce health care professionals’ cognitive load in stressful high-intensity situations, increase access to evidence-based information at the point of care, and standardize emergency clinical decision making [9,13,15]. However, CDSSs have not been universally adopted because of the perceptions of clinicians and administrators that they are expensive, lack usability, and that their content is difficult to adapt to local context [6,16-23].

Wikis can be an innovative component of a CDSS, which may support their implementation by addressing local adaptability issues and costs [24]. Wikis are collaborative writing technologies [25] that allow the creation of interactive, rapidly expanding, and low-cost knowledge databases [22,26]. Wikis allow people not only to consume content but also to produce and edit knowledge [27,28]. In the health care context, wikis (eg, WikEM [29] and Canadian Computerized Provider Order Entry Toolkit [30]) allow knowledge users (eg, physicians and administrators) to create and maintain a knowledge base that can quickly adapt to the local context at a low cost [26,31]. Wikis offer several advantages, including an immediate access to new or updated knowledge and interinstitutional integration [10-14,26]. As such, a wiki can act as the organizational memory of learning organizations where multiple interprofessional stakeholders can create, update, and share knowledge that promotes best practices [1,26,31-33]. This knowledge can take the form of explicit knowledge tools (eg, protocols, order sets, reminders, care pathways, and decision aids) created to support decision making by clinicians and patients based on the best evidence available from rigorous clinical practice guidelines and systematic reviews [34-37]. Relying on wiki capacities to manage knowledge, some health organizations have begun using wiki-based knowledge tools to support the implementation of best practices [19,25,38-44]. Given the potential of wiki-based knowledge tools to improve clinical practice, it is important to understand the factors that would contribute to their uptake by health care professionals.

### Conceptual Framework

**Figure 1:**
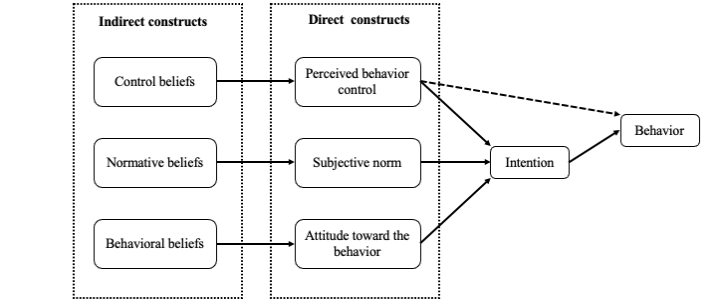
Theory of planned behavior model.

The theory of planned behavior (TPB; Figure 1) has been successfully applied [9,45-47] to study a wide range of health care professionals’ behaviors. A recent systematic review has shown that internet-based interventions based on the TPB tend to exert substantial effects on behavior [9]. According to Ajzen [48], the adoption of a new behavior is predicted by the person’s intention to engage in that behavior. Intention depends on three main behavioral determinants (direct constructs)—attitude, subjective norm (SN), and perceived behavioral control (PBC). Ajzen [48] also identifies three types of beliefs (indirect constructs) that may influence behavioral determinants—behavioral, normative, and control beliefs (Figure 1). For example, a clinician’s intention to use a wiki-based knowledge tool could be strongly influenced by the barriers to access the wiki in the workplace (control belief), a departmental chief not supporting the use of the wiki (normative belief), or a belief that the wiki will help access up-to-date clinical evidence (behavioral belief) [31]. von Haeften et al [49] affirm that to change an intention (and its corresponding behavior), it is necessary to identify and change the determinants of that intention.

According to the TPB as described above, we hypothesize that we can identify the salient beliefs that determine the EPs’ and ACHPs’ intention to use wiki-based knowledge tools. Moreover, based on our previous qualitative exploration of EPs and ACHPs beliefs demonstrating different beliefs for each professional group [1], we hypothesized that the salient beliefs influencing the intention to use wiki-based knowledge tools would be different for EPs and ACHPs. Identifying the beliefs that have the strongest influence on EP and ACHP intentions will allow us to build a theory-based intervention specific to each professional group for promoting the use of wiki-based knowledge tools in trauma centers. The ultimate goal of such an intervention is to improve the quality of care within learning health organizations [1,40].

## Methods

### Study Design, Setting, Population, and Protocol

We conducted our survey using 2 previously developed and tested TPB questionnaires to evaluate EPs’ and ACHPs’ intention to use wiki-based knowledge tools [31,50] and report its results using the Checklist for Reporting Results of Internet E-Surveys [51-53] (Multimedia Appendix 1 [50]). These questionnaires were previously developed and tested in French with Quebec EPs and ACHPs and revealed adequate internal consistency and stability over time [31,50]. The TPB questionnaires aimed to identify the behavioral determinants that had the greatest influence on the intention to use wiki-based knowledge tools.

The study was conducted in 12 designated trauma centers [54], including 1 level I, 5 level II, and 6 level III trauma centers in the province of Quebec, Canada. Quebec is Canada’s second most populous province [55]. The trauma system in Quebec was launched in 1993 and involves an integrated continuum of care from rural community hospitals to urban trauma centers. This system relies on certified ACHPs and EPs who use standardized care protocols across the province. The trauma center designation levels are revised periodically with on-site visits according to the American College of Surgeons criteria [56]. Trauma care services in Quebec are based on transfer agreements between hospitals and a no-refusal transfer policy [57]. Level I, II, and III centers are designated trauma centers with varying levels of services being provided. Level I trauma centers are large, urban hospitals with 24×7 orthopedic, vascular, neurosurgical, and trauma surgery coverage, along with emergency and specialized intensive care services. Level II trauma centers offer full-time, year-round coverage of orthopedic and general surgeries and run an intensive care unit staffed by full-time certified intensivists and an ED staffed by certified EPs. Level III trauma centers offer full-time, year-round coverage of general surgery and partial coverage of orthopedic surgery; they run an ED staffed by general practitioners. They also have an intensive care unit, but they are not run by full-time certified intensivists [56,57]. For the purposes of this study, participants were EPs (excluding residents and medical students) and certified ACHPs (nurses, respiratory therapists, and pharmacists) involved in caring for patients with trauma. Professionals not involved in emergency trauma care were excluded from the study. We purposefully established our list of 12 participating centers based on their geographic location and trauma level of care to recruit a proportion of trauma centers across the province that would reflect the same province-wide proportion of level I, II, and III centers.

To recruit participants, we sent an email to the head physician, nurse, respiratory therapist, and pharmacist of each ED. We asked them to send our invitations to all their respective department members with a web-based link to an electronic survey (SurveyMonkey [58]). Questionnaires were available only in French. A 2-week reminder to complete the web-based survey was sent in the same way. A final reminder was sent after 4 weeks to all potential participants using a ready-to-print PDF version. In total, 266 EPs and 907 ACHPs from 12 Quebec trauma centers were invited to participate. Participants were offered an incentive to participate by offering the chance to win 1 of the 3 electronic tablets. Data were collected between February 2014 and June 2015.

Before responding to the survey, participants were asked to view a 6-minute video (described elsewhere [50]) about wiki-based knowledge tools in trauma care to help them better understand the behavior being investigated. Briefly, participants were shown 1 of the 4 videos that were created specifically for their profession, demonstrating the use of a wiki-based knowledge tool in a simulated trauma case. After watching the appropriate video, the participants filled out 1 of 2 questionnaires according to their profession: EPs filled out the questionnaire for EPs, whereas nurses, respiratory therapists, and pharmacists filled out the questionnaire for ACHPs.

This study was approved by the Research Ethics Committee at the Centre de Santé et Services Sociaux Alphonse-Desjardins as a multicenter research study and by the local ethics review board of each participating center, under the study protocol number MP-23-2014-222. All ED directors approved our project before sending our survey to their members. Participation in the study was voluntary, and the completion of the electronic and paper survey implied consent for participation. To ensure participant privacy and anonymity, no personal information, including internet protocol addresses, was collected.

### Measurements

The EP questionnaire comprised 45 items and the ACHP questionnaire comprised 43 items. Briefly, the questionnaires measured sociodemographic, and direct and indirect TPB constructs, as explained elsewhere [1,45,50]. For both questionnaires, the items were measured on a 7-point Likert scale ranging from 1 to 7 (eg, “strongly disagree” [score of 1] to “strongly agree” [score of 7] with “neither agree nor disagree” at the center [score of 4]). Both questionnaires contained 12 sociodemographic questions (eg, age, gender, profession, years of work experience, and previous experience of wiki use in either professional or personal life) and took approximately 10 minutes to complete. SurveyMonkey automatically collected the data for the web version in an Excel spreadsheet, and the responses were manually entered into a spreadsheet for the paper-based questionnaires.

### Data Analysis

Before commencing any statistical analyses, data were visually inspected for outliers and checked for normality. Descriptive statistics (means, SDs, and frequencies) summarized and compared demographic information and TPB variables for EP and ACHP participants. For each TPB construct with more than 2 questionnaire items, missing data on items were imputed by using the mean of the other items. The internal consistency of each TPB construct was verified using Cronbach *α* coefficients for constructs measured using three questionnaire items.

Bivariate analyses were performed between the outcome variable (intention) and the independent variables (demographic information and TPB constructs) using Pearson correlations and Student two-tailed *t* tests. For each type of participant (EP vs ACHP), we then performed a first linear regression model including only TPB direct constructs. We then used a backward approach to test the model adjustment with demographic variables (*P*<.10) [49]. Then, we calculated the proportion of variance (*R*^2^) explained by the model. Then, to identify significant underlying beliefs, we replaced significant direct constructs (PBC, SN*,* and attitude) that predicted professionals’ intention to use wiki-based knowledge tools with their associated indirect constructs ("control", "normative", and "behavioral beliefs"). Following a backward approach, we only retained significant beliefs (salient beliefs; *P*<.05). Linear regression assumptions were verified for all models. All analyses were performed using the statistical analysis SAS software (SAS Institute Inc) version 9.4 for Windows.

## Results

### Flow of Participants and Participants’ Characteristics

**Figure 2:**
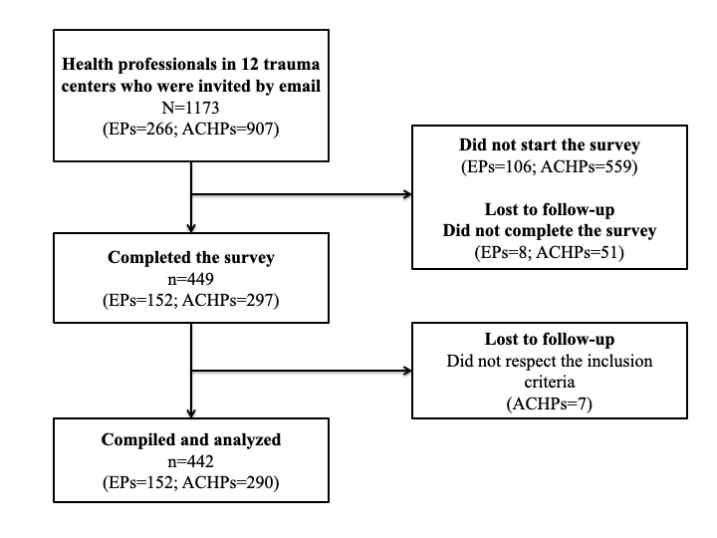
Flowchart of participants of the 12 designated trauma centers. ACHP: acute care health professional; EP: emergency physician.

The demographic characteristics of the participants are presented in Table 1, and their flowchart is presented in Figure 2. Overall, 57.1% (152/266) of EPs and 31.9% (290/907) of ACHPs responded to our survey from 12 trauma centers (level I, II, and III). Among the 442 participants, 337 (76.2%) were women. Their ages ranged from 21 to 69 years, with a mean of 37 (SD 9) years for EPs and 37 (SD 10) years for ACHPs. Among EPs, 49% (74/151) had a special competence in emergency medicine from the College of Family Physicians of Canada, 7.9% (12/151) were certified in emergency medicine as fellows of the Royal College of Physicians and Surgeons of Canada, and 43% (65/151) had no specific certification in emergency medicine. The 290 ACHPs comprised 3 groups of professionals: 196 (67.6%) were nurses, 61 (21%) were respiratory therapists, and 33 (11.4%) were pharmacists (Table 1).

**Table 1:** Baseline characteristics of participating emergency physicians and ACHPs^a^.

Variables				Emergency physicians		ACHPs
**Trauma center level, n (%)**						
	Level III		39 (25.7)		90 (31)	
	Level II		87 (57.2)		138 (47.6)	
	Level I		26 (17.1)		62 (21.4)	
**Age (years)^b^**						
	Value, mean (SD)		37 (9)		37 (10)	
	Value, min-max^c^		25-59		21-69	
**Clinical experience (years)^b^**						
	Value, mean (SD)		10 (8)		14 (10)	
**Gender^b^, n (%)**						
	Women		94 (62.3)		243 (84.1)	
	Men		57 (37.7)		46 (15.9)	
**Emergency medicine certification^b^, n (%)**						
	CCFP-EM^d^		74 (49)		N/A^e^	
	FRCPC^f^		12 (7.9)		N/A	
	No certification		65 (43)		N/A	
**ACHPs**						
	Nurses		N/A		196 (67.6)	
	Respiratory therapist		N/A		61 (21)	
	Pharmacist		N/A		33 (11.4)	

^a^ACHP: acute care health professional.

^b^Missing data: emergency physicians=1; acute care health professionals=1.

^c^Range.

^d^CCFP-EM: College of Family Physicians of Canada.

^e^N/A: not applicable.

^f^FRCPC: Fellows of the Royal College of Physicians and Surgeons of Canada.

### Descriptive Analysis of the Theoretical Constructs

For EPs, the internal consistency was adequate for all direct TPB constructs (Cronbach *α*=.76-.90). For ACHPs, the intention and attitude constructs had an appropriate internal consistency (Cronbach *α*=.85 and Cronbach *α*=.80, respectively). For PBC and SN constructs, one question was removed from each construct to obtain appropriate internal consistency. The results in Table 2 indicate that participants expressed a high intention (EPs: mean 5.68, SD 1.04; ACHPs: mean 5.49, SD 1.11; on a 7-point Likert scale) to use wiki-based knowledge tools. The PBC (EPs: mean 6.2, SD 0.93; ACHPs: mean 5.85, SD 1.39) was the highest rated direct construct in both groups. In addition, the SN was higher for ACHPs (mean 5.35, SD 1.08) than for EPs (mean 3.65, SD 1.3; *P*<.001).

**Table 2:** Descriptive analysis of the theoretical variables^a^.

Direct construct	Emergency physicians			ACHPs^b^			*P* value
	Value, mean (SD)	Cronbach *α*	Value, mean (SD)		Cronbach *α*		
Intention	5.68 (1.04)	.90	5.49 (1.11)		.85	.08	
PBC^c^	6.20 (0.93)	.79	5.85 (1.39)		.74	.002	
Subjective norm	3.65 (1.3)	.76	5.35 (1.08)		.58	<.001	
Attitude	6 (0.89)	.89	5.59 (0.89)		.8	<.001	

^a^All scores vary between 1 and 7.

^b^ACHP: acute care health professional.

^c^PBC: perceived behavioral control.

### Bivariate and Multivariable Analysis

#### Results for EPs

The matrix of correlations between direct model variables is presented in Table 3. All independent variables correlated significantly with intention (*r*=0.33-0.74). On the basis of the strong correlation between age and experience (Pearson correlation; *r*=0.88), only age was considered in the analysis. Bivariate analyses are presented in Multimedia Appendix 2. Among all demographic variables measured in the questionnaire, three associations with EPs’ intention were found to be significant (*P*<.10). Older EPs had a lower intention to use wiki-based knowledge tools in trauma centers (Pearson correlation; *r*=−0.14; *P*=.06) than younger EPs. Similarly, EPs certified as a Fellow of the Royal College of Physicians and Surgeons of Canada had a lower intention (mean 4.86, SD 1.42) to use a wiki-based knowledge tool (*P*=.02) than EPs without certification (mean 5.71, SD 0.94) or with a College of Family Physicians of Canada certification (mean 5.78, SD 1.01). Previous professional use of wikis was associated with an increased intention (mean 6.03, SD 0.803) in using wiki-based knowledge tools (*P*=.09).

**Table 3:** Correlation analysis for emergency physicians and ACHPs^a^.

Correlation analysis			Intention	PBC^b^	SN^c^	Attitude
**Pearson emergency physicians**						
	**Intention**					
		*r*	1	0.43	0.33	0.74
		*P* value	—^d^	<.001	<.001	<.001
	**PBC**					
		*r*	0.43	1	0.02	0.43
		*P* value	<.001	—	.84	<.001
	**SN**					
		*r*	0.33	0.02	1	0.15
		*P* value	<.001	.84	—	.06
	**Attitude**					
		*r*	0.74	0.43	0.15	1
		*P* value	<.001	<.001	.06	—
**Pearson ACHPs**						
	**Intention**					
		*r*	1	0.46	0.61	0.68
		*P* value	—	<.001	<.001	<.001
	**PBC**					
		*r*	0.46	1	0.31	0.36
		*P* value	<.001	—	<.001	<.001
	**SN**					
		*r*	0.61	0.31	1	0.55
		*P* value	<.001	<.001	—	<.001
	**Attitude**					
		*r*	0.68	0.36	0.55	1
		*P* value	<.001	<.001	<.001	—

^a^ACHP: acute care health professional.

^b^PBC: perceived behavioral control.

^c^SN: subjective norm.

^d^Not applicable.

The linear regression model with the TPB direct constructs and demographic variables indicated that all three direct TPB constructs were associated with the intention to use wiki-based knowledge tools (Table 4). This model, based on TPB direct constructs, explained 62% of the variance in the intention to use wiki-based knowledge tools. Attitude (β=.75) was the most important predictor of EP use of wiki-based knowledge tools to promote best practices in trauma care. None of the EPs’ sociodemographic variables remained significant in this model.

**Table 4:** Multiple linear regression analysis for emergency physicians and ACHPs^a^.

Variable		Estimated value of parameters (SE)	*P* value
**Emergency physicians’ final TPB^b^ model for direct constructs**			
	Intercept	−0.52 (0.43)	.24
	PBC^c^	0.16 (0.06)	.01
	SN^d^	0.19 (0.04)	<.001
	Attitude	0.75 (0.07)	<.001
**ACHPs’ final TPB model for direct constructs**			
	Intercept	−0.30 (0.29)	.30
	PBC	0.17 (0.03)	<.001
	SN	0.32 (0.05)	<.001
	Attitude	0.56 (0.06)	<.001
	Profession (respiratory therapist)	−0.42 (0.11)	.001
	Profession (pharmacist)	−0.11 (0.14)	.45
	Wiki for personal use	0.19 (0.09)	.03

^a^ACHP: acute care health professional.

^b^TPB: theory of planned behavior.

^c^PBC: perceived behavioral control.

^d^SN: subjective norm.

**Figure 3:**
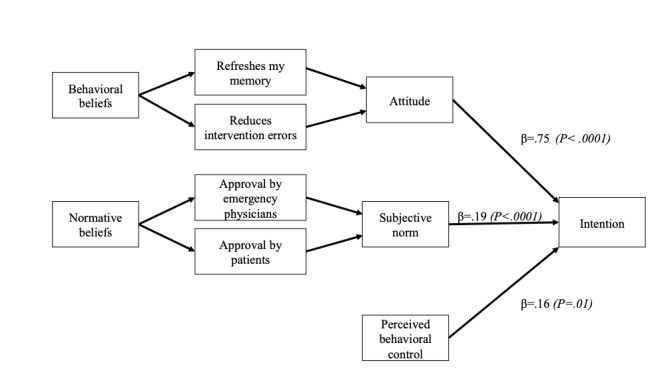
Emergency physicians’ final theory of planned behavior model with direct and indirect constructs (β weights and *P* values in parentheses).

To determine the salient beliefs for predicting EPs’ intention to use wiki-based knowledge tools, all significant TPB direct constructs in the first linear regression model were replaced with their associated beliefs in a second regression model. The final model (Table 5) identified significant normative beliefs ("approval from EPs" and "patients") and two behavioral beliefs (wiki-based knowledge tools "refresh my memory" and "reduce intervention errors"; Multimedia Appendices 3 and 4). Figure 3 presents a summary of all the constructs that influence EPs’ intention to use wiki-based knowledge tools.

**Table 5:** Salient belief analysis for both emergency physicians and ACHPs^a^.

Variable		Estimated value of parameters (SE)	*P* value
**Emergency physicians**			
	Intercept	−0.92 (0.61)	.13
	Support by emergency physicians	0.27 (0.07)	.001
	Support by patients	0.19 (0.04)	<.001
	Refreshes my memory	0.43 (0.09)	<.001
	Reduces intervention errors	0.21 (0.08)	.009
**ACHPs**			
	Intercept	0.80 (0.37)	.03
	Time constraints	0.14 (0.03)	.001
	Supported by people less comfortable with information technology	0.10 (0.04)	.01
	Supported by my hospital trauma team	0.32 (0.05)	<.001
	Access to evidence	0.29 (0.05)	<.001

^a^ACHP: acute care health professional.

#### Results for ACHPs

For ACHPs, the matrix of correlations between the direct constructs is shown in Table 3. All independent variables correlated significantly with intention (*r*=0.46-0.68). Correlations between the independent variables were also important (*r*=0.31-0.55). Among all demographic variables measured in the ACHP questionnaire, three significant associations with ACHP intentions were found. ACHPs who did not have access to a computer had a lower intention to use wiki-based knowledge tools than ACHPs with computer access (*P*=.04). Moreover, ACHPs who previously used a wiki in their workplace had a higher intention to use wiki-based knowledge tools (*P*=.009). ACHPs in level I hospitals had a higher intention to use wiki-based knowledge tools than ACHPs in level II and III hospitals (*P*<.001). Otherwise, no significant bivariate associations were found with the type of profession (*P*=.36) or a previous personal use of a wiki (*P*=.13). Bivariate analyses are shown in Multimedia Appendix 5.

The results of the multiple linear regression model using the direct TPB constructs and demographic variables indicated that all three direct constructs were significantly associated with the intention to use wiki-based knowledge tools (PBC: *P*<.001; SN: *P*<.001; attitude: *P*<.001). Two sociodemographic variables remained significant in this model: profession and previous use of a wiki for personal use. The final model is presented in Table 4. This model explains 60% of the variance in the intention to use wiki-based knowledge tools. Attitude (β=.56) was the most important predictor of ACHPs’ use of wiki-based knowledge tools to promote best practices in trauma care centers.

**Figure 4:**
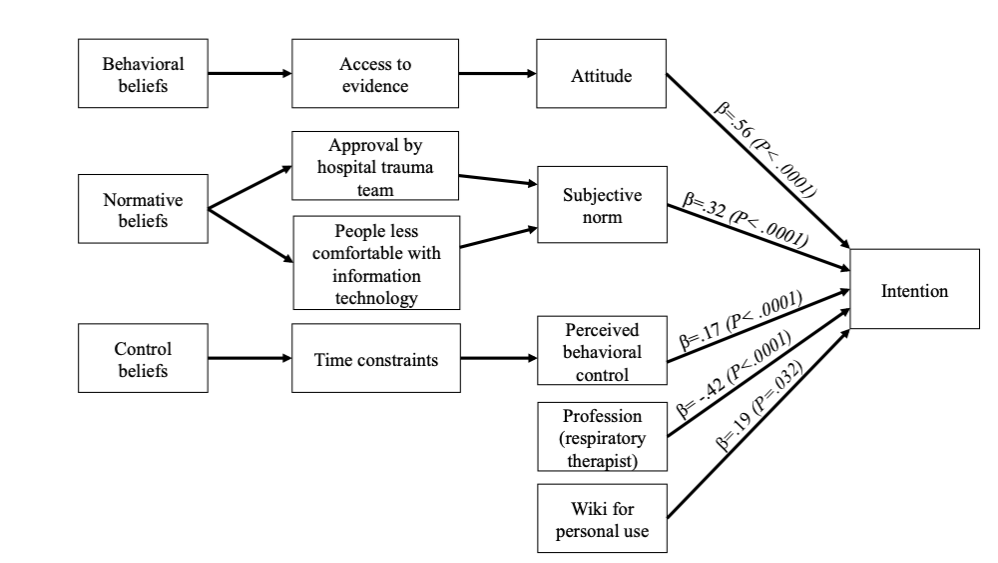
Acute care health professionals’ final theory of planned behavior model with direct and indirect constructs (β weights and *P* values in parentheses).

To identify the salient beliefs that predict ACHPs’ intention to use wiki-based knowledge tools, all significant TPB direct constructs were replaced with their associated indirect constructs (beliefs) in a second linear regression model. The final model obtained using the backward selection approach is presented in Table 5. We found that two normative beliefs ("people less comfortable with information technology" and "my hospital trauma team"), one control belief ("I would use wikis even if I had time constraints"), and one behavioral belief ("If I used a wiki, it would give me access to evidence") were significant, as shown in Multimedia Appendices 3 and 6. Figure 4 presents a summary of all the constructs that influence ACHPs’ intention to use wiki-based knowledge tools.

## Discussion

### Principal Findings

This study identified the salient beliefs in emergency health care professionals (EPs and ACHPs) that can predict the intention to use wiki-based knowledge tools for promoting best practices in trauma care centers. With these results, we can better understand how wiki-based knowledge tools can be used to increase evidence-based practices in trauma care and how to maximize the use and benefits of wiki-based knowledge tools. This will inform the construction of novel educational interventions to address specific beliefs to increase EPs and ACHPs use a wiki-based knowledge tool.

The research reported here provides data from a system-wide survey conducted in a wide range of trauma centers that increases its applicability to promote the implementation of best practices in trauma care across the full range of the trauma continuum. Other theory-based investigations [1,59-62] have been conducted to explore behaviors with respect to contributing to a wiki or using wiki content in contexts other than quality improvement in health care, but this study identifies the specific behavioral determinants needed to address in the context of a health care wiki-based quality improvement intervention. Overall, the intention to use wiki-based reminders to support best practice implementation was high for both EPs and ACHPs. These findings are similar to those reported by Gupta et al [41] and Wright et al [19] regarding the use of wikis in the context of the collaborative design of an asthma action plan and the sharing of clinical decision support content with Web 2.0, respectively. Other researchers have found lower expressed intentions to use wiki-based information in various contexts [63,64]. A randomized controlled trial comparing an in-person nominal group approach with an internet-based wiki-inspired alternative for engaging stakeholders in chronic kidney disease research prioritization identified a low correlation in rankings as compared with the wiki groups, with less satisfaction and perceptions of active engagement [64]. We believe that our positive results regarding EPs’ and ACHPs’ intentions to use our wiki model probably reflect our participants’ trust in the expert-created content model we proposed in our videos as opposed to a model of layperson-created content such as Wikipedia.

In our study, age, gender, years of experience, access to a computer with internet, the frequency of using another professional wiki, previous wiki edition experience, or trauma committee membership did not have any influence on either EPs’ or ACHPs’ intention to use wiki-based knowledge tools for promoting trauma care best practices. We found that the ACHP profession type was related to the intention to use wiki-based knowledge tools with pharmacists and respiratory therapists, both having a lower intention to use wiki-based knowledge tools compared with nurses. Conversely, the previous use of a wiki for personal reasons increased the ACHPs’ intention to use a wiki-based knowledge tool. Our analysis showed that the level of the trauma center did not influence the intention to use wiki-based knowledge tools.

We also found that ACHPs were a heterogeneous group and had different behavioral determinants toward using wiki-based knowledge tools. The ACHPs were nurses, respiratory therapists, and pharmacists, all of whom had different clinical tasks. We suggest that future studies should consider the particularities of each profession. We have demonstrated that both EPs and ACHPs have a good perception of their ability (PBC) to use a wiki-based knowledge tool. In other words, in general, they feel confident that they will be able to use this type of technology. However, our salient belief analysis showed that some ACHP subgroups feel less comfortable with information technology. Other studies have also shown that certain health professionals such as nurses express the need for educational programs to enhance their level of comfort with information technology [65-67] and with wiki technology [25] in particular.

ACHPs also perceived time constraints as a potential barrier to the use of wiki-based knowledge tools. Although time constraint was not a salient belief for EPs in our study, this contrasts with earlier studies that have identified time constraints as an important control belief in technology adoption [9,45,68] and in other contexts as well [9,69] for EPs and ACHPs alike. Given the tight time constraints associated with trauma care, ACHPs appear to appreciate brevity and efficiency [13,15]. Although our study did not show time constraints as a significant salient belief for EPs, we do not believe EPs will differ from ACHPs in this aspect based on previous studies [25]. Consequently, interventions targeting these control beliefs will most likely need to be oriented toward showing the efficiency of using wiki-based knowledge tools to improve trauma care decision making for EPs and ACHPs alike.

EPs and ACHPs are also more likely to engage in using wiki-based knowledge tools if they know that using such tools will refresh their memory, give them access to evidence-based knowledge tools, and reduce intervention errors. Consequently, educational interventions targeting these behavioral beliefs will have to show that using a wiki-based knowledge tool can help EPs and ACHPs reduce medical errors and remind them about the best evidence to use [9,27,69]. Although our results indicate that EPs feel less social pressure to use wiki-based knowledge tools than ACHPs, both EPs and ACHPs are both more likely to engage in using wiki-based knowledge tools if they feel supported by their colleagues and their patients. Therefore, we could develop common behavioral change techniques that support the collaborative use of wiki-based knowledge tools, interprofessional communication, and local champions to lead the implementation of wiki-based reminders promoting practice change. Considering the value EPs place in support from patients, involving patient partners could also support using a wiki-based reminder system. The existing recommendations for patient-oriented research could help in engaging patients and clinicians in a collaborative quality improvement platform [70].

Our results also indicate that both ACHPs and EPs share the need for support from their peers (other EPs and trauma teams). This means that a common intervention targeting both EPs and ACHPs in trauma teams could improve the use of wiki-based knowledge tools. Interprofessional collaboration has been proposed as an important facilitator in the implementation of best trauma care practices [71,72].

This study adds to the understanding of using wiki-based knowledge tools to support the implementation of best practices in trauma care by using the TPB. In terms of the significance of the variables, our results are similar to those presented in previous studies that identified barriers and facilitators. For example, others have shown that the scientific quality of information resources [16,45] influences their use. We also found that wiki-based knowledge tool use will also be influenced by access to high-level evidence (ACHPs) and potentially reduce intervention errors (EPs). The analytical strategy used in this study provides scientific evidence to identify the most important determinants of EPs’ and ACHPs’ intentions to design an intervention aimed at promoting the use of wiki-based knowledge tools. We found that EPs’ and ACHPs’ intention to use wiki-based knowledge tools can be predicted by the three direct TPB constructs—attitude toward the behavior, SN, and PBC. We have also identified the salient beliefs that will help us develop a theory-based training program to promote the use of wiki-based knowledge tools in trauma care centers for EPs and ACHPs [40,73]. These salient beliefs will also inform the development of interventions that support the implementation of future wiki-based knowledge tools for other acute care contexts, such as optimal ED elder care [74] and pandemic knowledge management [75].

### Limitations

This study has several limitations. First, the principal limitation of our study is not being able to measure the actual behavior. This is a preliminary study that will help us construct a wiki system containing knowledge tools to promote best practices in trauma care that will consider all the identified behavioral determinants [12,45]. According to the TPB, intention is assumed to be an immediate antecedent of behavior, and measures of behavioral intention are frequently used as a proxy for actual behavior [45].

Second, this study was conducted in 12 publicly funded health organizations in the province of Quebec, a French-speaking region of Canada. Thus, the results may not be generalizable to other types of organizations and other settings. However, given the strong predictive power of the theoretical model, we believe that our approach can inform similar studies in other locations.

Third, we did not separate specific beliefs for each ACHP category. However, our results suggest that ACHP characteristics need to be considered while evaluating the intention to use wiki-based knowledge tools. We suggest that future studies should consider the particularities of each type of health professional. Finally, there are other limitations related to our survey methodology. Our study involved voluntary participation, which may have introduced a selection bias. Study participants may have had more experience or a stronger intention to use wiki-based knowledge tool than nonparticipants. For this reason, it is possible that a social desirability bias positively influenced our results. Moreover, this survey was conducted in 2014 and 2015. Although this does not affect the internal validity of our results, it might potentially affect the applicability of the paper in today’s context as technology and its acceptance may have evolved. Furthermore, our linear regression model for ACHPs seems to be affected by two variables (profession and the previous use of a wiki for personal use) with a small confounding effect. Unbalanced data between categorical modalities of these two variables may have attenuated the true relation with intention in bivariate analyses.

### Conclusions

This study allows us to better understand how a wiki-based knowledge tool can be used to increase evidence-based practices and maximize their benefits. This will be useful in constructing an implementation intervention that supports the best practices in trauma care. This study contributes to knowledge translation and organizational learning by proposing a strong theoretical basis to assess the determinants of using wiki-based knowledge tools in trauma care centers. Future studies are needed to assess the impact of using wiki-based knowledge tools on health care professionals’ knowledge, attitudes, skills, and behaviors in practice as well as to address the barriers to their use, to find ways to ensure the quality of their content, to foster contributions, and to make these tools effective knowledge translation tools for different stakeholders.

## Appendix

Multimedia Appendix 1 Checklist for Reporting Results of Internet E-Surveys guideline report.

Multimedia Appendix 2 Bivariate analysis for emergency physicians.

Multimedia Appendix 3 Emergency physicians’ and acute care health professionals’ indirect constructs.

Multimedia Appendix 4 Emergency physicians’ salient belief analysis.

Multimedia Appendix 5 Bivariate analysis for acute care health professionals.

Multimedia Appendix 6 Acute care health professionals’ salient beliefs’ analysis.

## Abbreviations

ACHP: acute care health professional
CDSS: clinical decision support system
ED: emergency department
EP: emergency physician
PBC: perceived behavioral control
SN: subjective norm
TPB: theory of planned behavior
