# Supplementary material for: Intention to Use Wiki-Based Knowledge Tools: Survey of Quebec Emergency Health Professionals
Source: JMIR Med Inform. 2021 Jun 18;9(6):e24649. doi: 10.2196/24649 (PMC8277401; doi:10.2196/24649)
Supplement: Multimedia Appendix 1 [file medinform_v9i6e24649_app1.docx]

**CHERRIES GUIDELINE REPORT**

# A measure of Quebec Emergency Health Professionals' Intention to Use Wiki-based Knowledge Tools

Patrick Archambault MD MSc FRCPC*^1,2,3,4^, Stéphane Turcotte MSc^3^, Pascal Y. Smith PhD^3^, Kassim Said Abasse PhD^3,4,5,6^, Catherine Paquet PhD^7^, André Côté PhD^4,5,6^, Dario Gomez MSc^9^, Hager Khechine PhD^9^, Marie-Pierre Gagnon PhD^4,8^, Melissa Tremblay MD^10^, Nicolas Elazhary, MD^10^, France Légaré MD PhD CFPC ^1,4,6^, on behalf of the Wiki-based Knowledge Tool Investigators

**Author’s Affiliation**

^1^Department of Family Medicine and Emergency Medicine, Faculty of Medicine, Université Laval, Québec, QC, Canada.

^2^Department of Anesthesiology and Critical Care Medicine, Intensive Care Division, Faculty of Medicine, Université Laval, Québec, QC, Canada.

^3^Centre intégré en santé et services sociaux de Chaudière-Appalaches, Lévis, QC, Canada.

^4^VITAM, Centre de recherche en santé durable, Université Laval, Quebec, QC, Canada.

^5^Département de management, Faculté des sciences de l’administration, Université Laval, Quebec, QC, Canada

^6^Centre de recherche du CHU de Quebec, Université Laval, Quebec, QC, Canada.

^7^Département de Marketing, Faculté des sciences de l’administration, Université Laval, Quebec, QC, Canada.

^8^Faculté des sciences infirmières, Université Laval, Quebec, QC, Canada

^9^Département de systèmes d’information organisationnels, Faculté des sciences de l’administration, Université Laval, Quebec, QC, Canada

^10^Départment of Family Medicine and Emergency Medicine, Faculty of Medicine, Université de Sherbrooke, Sherbrooke, QC, Canada

***Corresponding Author**

Patrick Archambault MD, MSc, FRCPC

CISSS Chaudière-Appalaches (Secteur Alphonse-Desjardins)

143 Rue Wolfe, Lévis, QC Room 4817

G6V 3Z1 CANADA

Cell: 418-955-2552

Phone: 418-835-7121 (ext. 13905)

E-mail:[patrick.m.archambault@gmail.com](mailto:patrick.m.archambault@gmail.com)

**Table 1: Checklist for Reporting Results of Internet E-Surveys (CHERRIES)**

| **Item Category** | **Checklist Item** | **Verification of Item in the Study** | **Validation** |
| --- | --- | --- | --- |
| Design | Describe survey design | This study took place in 12 designed trauma centers in the province of Quebec, Canada. Participants were emergency physicians (EPs) (excluding residents and medical students) and acute care healthcare professionals (ACHPs) (nurses, respiratory therapists and pharmacists). A total of 1173 health professionals (EPs=266 and ACHPs=907) were invited by email. Among the 1173 invited health professionals, 152 EPs and 290 ACHPs completed the questionnaire. | OK |
| Institutional Review Board (IRB) approval and informed consent process | IRB approval | This study was approved by the Research Ethics Committee at the Centre de santé et de services sociaux Alphonse-Desjardins, as a multicenter research study and by the local ethics review board of each participant center, under the study protocol number (**MP-23-2014-222)**. | OK |
|  | Informed consent | All emergency department (ED) directors approved our project before sending out our survey to their members. Participation in this study was voluntary and completion of the electronic and paper survey implied consent to participate. | OK |
|  | Data protection | The study followed guidelines of the Declaration of Helsinki for humans. The data collected by this questionnaire were anonymous and it were treated with confidentiality and were not associated with their institution in any circumstances, when the results were made public. | OK |
| Development and pretesting | Development and testing | Development and validation of questionnaires had been completed in a previously published study in JMIR ResProtoc, 2014[1]. | OK |
| Recruitment process and description of the sample having access to the questionnaire | Open survey versus closed survey | This was a closed survey using a validated Theory of Planned Behavior (TPB) questionnaire [1] to evaluate EPs and ACHPs’ intention to use wiki-based knowledge tools. | OK |
|  | Contact mode | To recruit participants, we sent an email to the head physician, nurse, respiratory therapist and pharmacist of each emergency department. We asked them to send our invitation to all their respective department members with an online link. | OK |
|  | Advertising the survey | This study used the head physician, nurse, respiratory therapist and pharmacist of 12 Trauma Care centers including 1 Level I, 5 Level II and 6 Level III to inform their respective department members. | OK |
| Survey administration | Web/E-mail | We sent an email to the head physician, nurse, respiratory therapist and pharmacist of each emergency department. We asked them to send our invitation to all their respective department members with an online link. | OK |
|  | Context | This study used an electronic online survey platform (SurveyMonkey). A link directly to the survey was provided. Questionnaires were only available in French. | OK |
|  | Mandatory/voluntary | This study was based on voluntary participation. | OK |
|  | Incentives | Participants were offered an incentive to participate by offering the chance to win one of three electronic tablets (valued at 700 CAN$). | OK |
|  | Time/Date | Data collection was undertaken between February 2014 and June 2015. | OK |
|  | Randomized of items or questionnaires | We did not apply any randomized items or questionnaires, however we used a structured questionnaire which was previously validated and published in JMIR ResProtoc [1]. | N/A |
|  | Adaptive questioning | This study did not use any form of adaptive questioning, however we used a validated questionnaires previously published in JMIR ResProtoc [1] | N/A |
|  | Number of Items | The validated EP questionnaire included 45 items and the ACHP questionnaire included 43 items. | OK |
|  | Number of screens (pages) | The number of screens was 13 pages. | OK |
|  | Completeness check | We did not use any form of completeness check before submitting the questionnaire. | N/A |
|  | Review step | The review step was not applied in this study. | N/A |
| Response rates | Unique site visitor | We have not documented the number of visits to our site. But at the same time, our site was not open to the public and therefore less relevant as a criterion of validity. We were not allowed to track IP addresses nor the use of cookies, nor to collect data. | N/A |
|  | View rate (Ratio of unique survey visitors / unique site visitors) | As mentioned above, we did not document the number of visits to our SurveyMonkey because we were not allowed to track IP addresses, nor use cookies to collect data. | N/A |
|  | Participation rate (Ratio of unique visitors who agreed to participate / unique first survey page visitors) | The participation rate was 43% (508/1173) with 60% (160/266) for EPs and 38% (348/907) for ACHPs. In addition, EPs’(Mean±SD) Age was (37±9) years old and ACHPs’ (Mean ± SD) Age was (37±10) years old. | OK |
|  | Completion rate (Ratio of users who finished the survey/users who agreed to participate) | Completion rate was 38% (442/1173) with 57% (152/266) for EPs and 32% (290/907) for ACHPs. | OK |
| Preventing multiple entries from the same individual | Cookies used | No cookies were used because in some EDs, the survey was available on a single computer allowing multiple individuals to reply to our survey from the same ED. | N/A |
|  | IP check | No IP check was performed. This was prohibited by our Ethics committee. | N/A |
|  | Log file analysis | We did review all participants in the exported Excel sheet and made sure we did not have any duplicate data entries. | OK |
|  | Registration | Unique IDs were given for each participant. Each individual only completed the survey once. | OK |
| Analysis | Handling of incomplete questionnaires | Incomplete questionnaires were handled as explained in the analysis section: " For each TPB construct with more than two questionnaire items, missing data on items were imputed by the mean of the other items. Internal consistency of each TPB construct was verified using Cronbach's alpha coefficients for constructs measured by three questionnaire items and Pearson correlations for the two constructs with only two items." Charts that contained too many missing data, were excluded from our analysis as presented in our Flow Chart. | OK |
|  | Questionnaires submitted with an atypical timestamp | Questionnaires that were not completed were removed as presented in our Flow Chart. | OK |
|  | Statistical correction | Statistical correction was applied as described in Analysis section. | OK |

**Reference**

1. Archambault PM, Gagnon S, Gagnon M-P, Turcotte S, Lapointe J, Fleet R, Côté M, Beaupré P, Le Sage N, Émond M: **Development and validation of questionnaires exploring health care professionals' intention to use wiki-based reminders to promote best practices in trauma**. *JMIR research protocols* 2014, **3**(4):e50.
