# Supplementary material for: Intention to Use Wiki-Based Knowledge Tools: Survey of Quebec Emergency Health Professionals
Source: JMIR Med Inform. 2021 Jun 18;9(6):e24649. doi: 10.2196/24649 (PMC8277401; doi:10.2196/24649)
Supplement: Multimedia Appendix 2 [file medinform_v9i6e24649_app2.docx]

# Multimedia Appendix 2: Bivariate analysis for emergency physicians (EPs)

## Variables: Age - Experience - Intention

**Descriptive statistics**

| Variable | N | Mean | SD | Sum | Min | Max |
| --- | --- | --- | --- | --- | --- | --- |
| Age | 151 | 37.31788 | 8.53961 | 5635 | 25.00000 | 59.00000 |
| Experience | 151 | 9.77099 | 8.23483 | 1475 | 0 | 34.00000 |
| Intention | 152 | 5.67763 | 1.03821 | 863.00000 | 2.00000 | 7.00000 |

**Pearson correlation coefficient; Proba > |r| sous H0: Rho=0**

**Number of observations**

|  | Age | Experience | Intention |
| --- | --- | --- | --- |
| Age | 1.00000  151 | 0.88396 <.0001  151 | -0.14826 0.0693 151 |
| Experience | 0.88396 <.0001 151 | 1.00000  151 | -0.04829 0.5560 151 |
| Intention | -0.14826 0.0693 151 | -0.04829 0.5560 151 | 1.00000  152 |

**Descriptive statistics by Sex and Mean comparison**

| Var Name | Gender | N | Mean | SD | 95% CI | Median | IQR | Min | Max | P-value* |
| --- | --- | --- | --- | --- | --- | --- | --- | --- | --- | --- |
| Intention | Female | 94 | 5.762 | 1.028 | (5.552; 5.973) | 6 | (5.333; 6.667) | 2 | 7 | 0.189 |
|  | Male | 57 | 5.532 | 1.056 | (5.252; 5.812) | 6 | (5; 6) | 2.667 | 7 |  |

***Test de student**

**Descriptive statistics by Certification REP and Mean comparison**

| Var Name | Certification Rep | n | Mean | SD | 95% CI | Median | IQR | Min | Max | P-value* |
| --- | --- | --- | --- | --- | --- | --- | --- | --- | --- | --- |
| Intention | No certification | 65 | 5.708 | 0.942 | (5.474; 5.941) | 6 | (5; 6) | 2.333 | 7 | 0.016 |
|  | Yes, CCFP-EM | 74 | 5.779 | 1.013 | (5.545; 6.014) | 6 | (5.333; 6.333) | 2 | 7 |  |
|  | Yes, FRCPC | 12 | 4.861 | 1.417 | (3.96; 5.762) | 5 | (4.5; 5.667) | 2 | 7 |  |

*ANOVA

**Descriptive statistics by Wiki for professional use and Mean comparison**

| Var Name | WikiProfRep | N | Mean | SD | 95% CI | Median | IQR | Min | Max | P-value* |
| --- | --- | --- | --- | --- | --- | --- | --- | --- | --- | --- |
| Intention | Yes | 22 | 6.03 | 0.803 | (5.674; 6.386) | 6 | (5.333; 6.667) | 4.667 | 7 | 0.085 |
|  | No | 130 | 5.618 | 1.064 | (5.433; 5.803) | 6 | (5; 6) | 2 | 7 |  |

*Test de student

**Descriptive statistics by Wiki for personal use and Mean comparison**

| Var Name | WikiPersoRep | N | Mean | SD | 95% CI | Median | IQR | Min | Max | P-value* |
| --- | --- | --- | --- | --- | --- | --- | --- | --- | --- | --- |
| Intention | Yes | 85 | 5.737 | 0.993 | (5.523; 5.952) | 6 | (5.333; 6.333) | 2 | 7 | 0.427 |
|  | No | 67 | 5.602 | 1.095 | (5.335; 5.869) | 6 | (5; 6) | 2 | 7 |  |

*Test de student

**Descriptive statistics by Committee trauma participation and Mean comparison**

| Var Name | Committee Trauma | N | Mean | SD | 95% CI | Median | IQR | Min | Max | P-value* |
| --- | --- | --- | --- | --- | --- | --- | --- | --- | --- | --- |
| Intention | Yes | 30 | 5.778 | 1.146 | (5.35; 6.206) | 6 | (5.333; 6.667) | 2.333 | 7 | 0.557 |
|  | No | 122 | 5.653 | 1.014 | (5.471; 5.835) | 6 | (5; 6) | 2 | 7 |  |

*Test de student
