# Supplementary material for: Intention to Use Wiki-Based Knowledge Tools: Survey of Quebec Emergency Health Professionals
Source: JMIR Med Inform. 2021 Jun 18;9(6):e24649. doi: 10.2196/24649 (PMC8277401; doi:10.2196/24649)
Supplement: Multimedia Appendix 3 [file medinform_v9i6e24649_app3.docx]

## Multimedia Appendix 3: Emergency physicians’ and acute care health professionals’ indirect constructs

|  | EPs | ACHPs |
| --- | --- | --- |
| normative beliefs | my use of wiki would be supported by:   - emergency physicians - patients | my use of wiki would be supported by:   - the people less comfortable with information technology in my hospital and - by my hospital trauma team |
| behavioral beliefs | - refreshes my memory; - reduces intervention errors | - If I used a wiki, it would give me access to evidence |
| control beliefs |  | - I would use wikis even if I had time constraints |

##### 
