# Supplementary material for: Intention to Use Wiki-Based Knowledge Tools: Survey of Quebec Emergency Health Professionals
Source: JMIR Med Inform. 2021 Jun 18;9(6):e24649. doi: 10.2196/24649 (PMC8277401; doi:10.2196/24649)
Supplement: Multimedia Appendix 4 [file medinform_v9i6e24649_app4.ppt]

## Slide 1
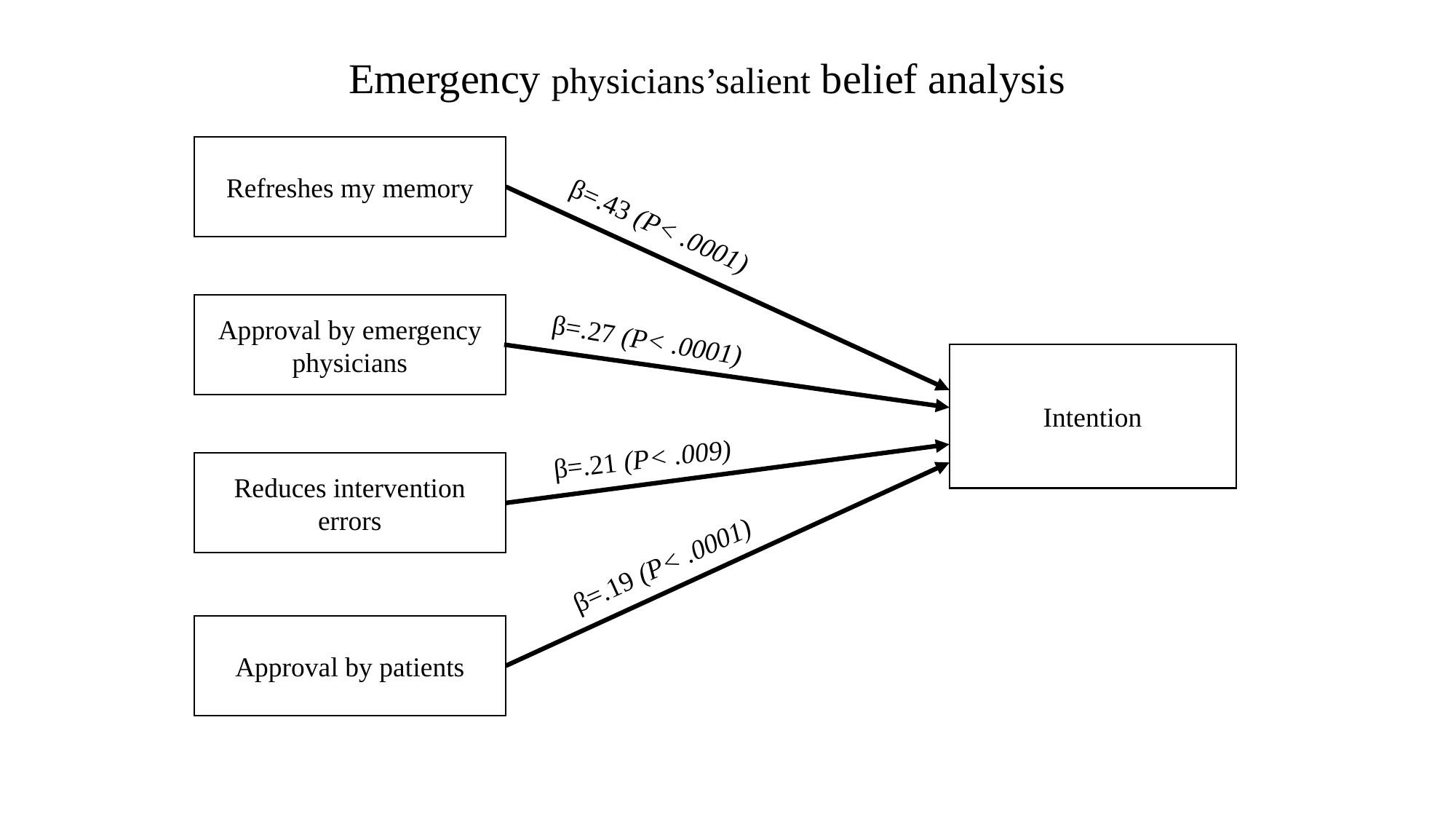

Emergency physicians’salient belief analysis
Refreshes my memory
β=.43 (P< .0001)
Approval by emergency physicians
β=.27 (P< .0001)
Intention
β=.21 (P< .009)
Reduces intervention errors
β=.19 (P< .0001)
Approval by patients
