# Supplementary material for: Intention to Use Wiki-Based Knowledge Tools: Survey of Quebec Emergency Health Professionals
Source: JMIR Med Inform. 2021 Jun 18;9(6):e24649. doi: 10.2196/24649 (PMC8277401; doi:10.2196/24649)
Supplement: Multimedia Appendix 5 [file medinform_v9i6e24649_app5.docx]

# Multimedia Appendix 5 : Bivariate analysis for acute care health professionals (ACHPs)

## Variables : Age - Experience - Intention

**Descriptive Statistics**

| Variable | N | Mean | SD | Median | Min | Max |
| --- | --- | --- | --- | --- | --- | --- |
| Age | 287 | 36.71429 | 9.89430 | 35.00000 | 21.00000 | 69.00000 |
| Experience | 290 | 13.74310 | 10.30774 | 12.00000 | 0.50000 | 65.00000 |
| Intention | 290 | 5.48793 | 1.11055 | 5.66667 | 1.66667 | 7.00000 |

**Pearson correlation coefficient; Proba > |r| sous H0: Rho=0;**

**Number of observations**

|  | Age | Experience | Intention |
| --- | --- | --- | --- |
| Age | 1.00000  287 | 0.87041 <.0001 287 | 0.04446 0.4530 287 |
| Experience | 0.87041 <.0001 287 | 1.00000  290 | 0.01582 0.7884 290 |
| Intention | 0.04446 0.4530 287 | 0.01582 0.7884 290 | 1.00000  290 |

**Pearson correlation coefficient Proba > |r| sous H0: Rho=0
Number of observations**

|  | Age | Experience | Intention |
| --- | --- | --- | --- |
| Age | 1.00000  287 | 0.88848 <.0001 287 | 0.03905 0.5100 287 |
| Experience | 0.88848 <.0001 287 | 1.00000  290 | -0.01433 0.8080 290 |
| Intention | 0.03905 0.5100 287 | -0.01433 0.8080 290 | 1.00000  290 |

**Descriptive Statistics by Sexe and Mean comparison**

| Var Name | Gender | N | Mean | SD | 95% CI | Median | Q1; Q3 | Min | Max | P-value |
| --- | --- | --- | --- | --- | --- | --- | --- | --- | --- | --- |
| Intention | Female | 243 | 5.475 | 1.098 | (5.337; 5.614) | 5.667 | (5; 6.333) | 1.667 | 7 | 0.7037 |
|  | Male | 46 | 5.543 | 1.195 | (5.188; 5.898) | 6 | (5; 6.333) | 2.333 | 7 |  |

**Descriptive Statistics by Profession and Mean comparison**

| Var Name | Variable | Profession | N | Mean | SD | 95% CI | Median | Q1; Q3 | Min | Max | P-value |
| --- | --- | --- | --- | --- | --- | --- | --- | --- | --- | --- | --- |
| Intention | Intention | Nurses | 196 | 5.522 | 1.118 | (5.365; 5.68) | 6 | (5; 6.333) | 1.667 | 7 | 0.3597 |
|  |  | Respiratory Therapist | 61 | 5.314 | 1.092 | (5.035; 5.594) | 5.333 | (4.667; 6) | 2 | 7 |  |
|  |  | Pharmacist | 33 | 5.606 | 1.098 | (5.217; 5.995) | 6 | (5.333; 6) | 2.333 | 7 |  |

**Descriptive Statistics by Acces computer and Mean comparison**

| Var Name | Variable | Acces Computer | N | Mean | SD | 95% CI | Median | Q1; Q3 | Min | Max | P-value |
| --- | --- | --- | --- | --- | --- | --- | --- | --- | --- | --- | --- |
| Intention | Intention | Yes | 243 | 5.54 | 1.082 | (5.403; 5.677) | 6 | (5; 6.333) | 1.667 | 7 | 0.0370 |
|  |  | No | 43 | 5.155 | 1.256 | (4.768; 5.542) | 5.333 | (4.667; 6) | 2 | 7 |  |

**Descriptive Statistics by WikiProfRep and Mean comparison**

| Var Name | Variable | WikiProfRep | N | Mean | SD | 95% CI | Median | Q1; Q3 | Min | Max | P-value |
| --- | --- | --- | --- | --- | --- | --- | --- | --- | --- | --- | --- |
| Intention | Intention | Yes | 55 | 5.836 | 1.122 | (5.533; 6.14) | 6 | (5.333; 6.667) | 2 | 7 | 0.0086 |
|  |  | No | 231 | 5.398 | 1.101 | (5.255; 5.54) | 5.667 | (5; 6) | 1.667 | 7 |  |

**Descriptive Statistics by WikiPersoRep and Mean comparison**

| Var Name | Variable | WikiPersoRep | N | Mean | SD | 95% CI | Median | Q1; Q3 | Min | Max | P-value |
| --- | --- | --- | --- | --- | --- | --- | --- | --- | --- | --- | --- |
| Intention | Intention | Yes | 108 | 5.61 | 1.107 | (5.398; 5.821) | 6 | (5; 6.333) | 1.667 | 7 | 0.1323 |
|  |  | No | 178 | 5.404 | 1.118 | (5.239; 5.57) | 5.667 | (4.667; 6) | 1.667 | 7 |  |

**Descriptive statistics by Level (Hospital) and Mean comparison**

| Var Name | Variable | Level | N | Mean | SD | 95% CI | Median | Q1; Q3 | Min | Max | P-value |
| --- | --- | --- | --- | --- | --- | --- | --- | --- | --- | --- | --- |
| Intention | Intention | Level 1 | 62 | 5.661 | 1.064 | (5.391; 5.931) | 6 | (5; 6.333) | 2 | 7 | <0.0001 |
|  |  | Level 2 | 138 | 5.45 | 1.067 | (5.271; 5.63) | 5.667 | (5; 6) | 1.667 | 7 |  |
|  |  | Level 3 | 90 | 5.426 | 1.204 | (5.174; 5.678) | 5.667 | (5; 6) | 1.667 | 7 |  |

**Descriptive statistics by Manual Response and Mean comparison**

| Var Name | Variable | Manuel response | N | Mean | SD | 95% CI | Median | Q1; Q3 | Min | Max | P-value |
| --- | --- | --- | --- | --- | --- | --- | --- | --- | --- | --- | --- |
| Intention | Intention | 0 | 257 | 5.5 | 1.097 | (5.365; 5.635) | 5.667 | (5; 6.333) | 1.667 | 7 | 0.6064 |
|  |  | 1 | 33 | 5.394 | 1.223 | (4.96; 5.828) | 5.667 | (5; 6) | 1.667 | 7 |  |

### Variables : Intention Contrôle – Norme - Attitude

**Descriptive Statistics**

| Variable | N | Mean | SD | Median | Min | Max |
| --- | --- | --- | --- | --- | --- | --- |
| Intention | 290 | 5.48793 | 1.11055 | 5.66667 | 1.66667 | 7.00000 |
| Control | 285 | 5.85439 | 1.39252 | 6.00000 | 1.00000 | 7.00000 |
| Norme | 284 | 5.35035 | 1.07639 | 5.50000 | 1.00000 | 7.00000 |
| Attitude | 289 | 5.59256 | 0.89214 | 5.75000 | 2.25000 | 7.00000 |

**Pearson correlation coefficient
Proba > |r| sous H0: Rho=0
Number of observations**

|  | Intention | Control | Norme | Attitude |
| --- | --- | --- | --- | --- |
| Intention | 1.00000  290 | 0.46459 <.0001 285 | 0.61113 <.0001 284 | 0.67761 <.0001 289 |
| Control | 0.46459 <.0001 285 | 1.00000  285 | 0.31443 <.0001 281 | 0.35838 <.0001 284 |
| Norme | 0.61113 <.0001 284 | 0.31443 <.0001 281 | 1.00000  284 | 0.55492 <.0001 283 |
| Attitude | 0.67761 <.0001 289 | 0.35838 <.0001 284 | 0.55492 <.0001 283 | 1.00000  289 |

**Pearson correlation coefficient**

**Proba > |r| sous H0: Rho=0**

**Number of observations**

|  | Intention | Control | Norme | Attitude |
| --- | --- | --- | --- | --- |
| Intention | 1.00000  290 | 0.59699 <.0001 285 | 0.59960 <.0001 284 | 0.65012 <.0001 289 |
| Control | 0.59699 <.0001 285 | 1.00000  285 | 0.40391 <.0001 281 | 0.47531 <.0001 284 |
| Norme | 0.59960 <.0001 284 | 0.40391 <.0001 281 | 1.00000  284 | 0.52939 <.0001 283 |
| Attitude | 0.65012 <.0001 289 | 0.47531 <.0001 284 | 0.52939 <.0001 283 | 1.00000  289 |
