# Supplementary material for: Intention to Use Wiki-Based Knowledge Tools: Survey of Quebec Emergency Health Professionals
Source: JMIR Med Inform. 2021 Jun 18;9(6):e24649. doi: 10.2196/24649 (PMC8277401; doi:10.2196/24649)
Supplement: Multimedia Appendix 6 [file medinform_v9i6e24649_app6.ppt]

## Slide 1
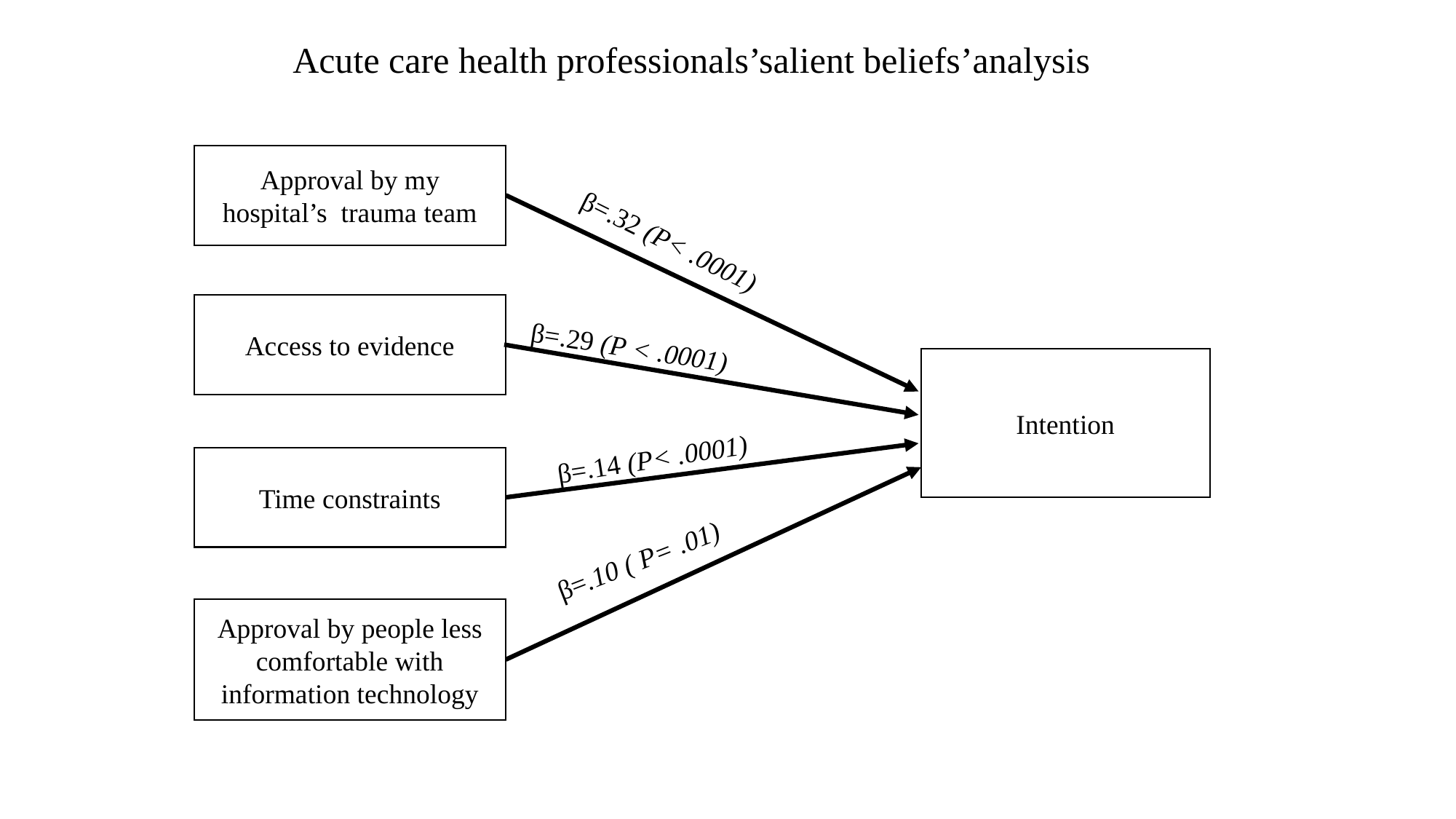

Acute care health professionals’salient beliefs’analysis
Approval by my hospital’s trauma team
β=.32 (P< .0001)
Access to evidence
β=.29 (P < .0001)
Intention
β=.14 (P< .0001)
Time constraints
β=.10 ( P= .01)
Approval by people less comfortable with information technology
